# Rev-Free HIV-1 Gene Delivery System for Targeting Rev-RRE-Crm1 Nucleocytoplasmic RNA Transport Pathway

**DOI:** 10.1371/journal.pone.0028462

**Published:** 2011-12-02

**Authors:** Narasimhachar Srinivasakumar

**Affiliations:** Division of Hematology/Oncology, Department of Internal Medicine, Saint Louis University, Saint Louis, Missouri, United States of America; Beckman Research Institute of the City of Hope, United States of America

## Abstract

The use of RNA transport elements from different viruses can provide novel attributes to HIV-1-based gene delivery systems such as improved safety or Rev independence. We previously described an HIV-1 based gene delivery system that utilized the simian immunodeficiency virus Rev-response element (RRE) in place of the HIV-1 RRE. Despite the use of Rev for the production of vector stocks, we showed the utility of this system for delivery of Rev M10, a dominant-negative mutant of HIV-1 Rev, into T-cells. Here, we investigated the use of RNA transport elements from Mason-Pfizer monkey virus or MPMV for the creation of high-titered Rev-free HIV-1-based packaging systems. The HIV-1 *gag/pol* expression constructs containing one or more copies of MPMV constitutive RNA transport element (CTE) were used to package similarly modified gene-transfer vectors in the presence or absence of Rev. An inverse correlation between the number of CTE modules and Rev dependency was noted for vector stock production. While packaging systems containing multiple CTEs were resistant to exogenously expressed Rev M10, the titers of vectors encoding Rev M10 were nevertheless reduced in comparison to vectors encoding only green fluorescent protein (GFP). In contrast, a gene transfer vector encoding the Rev M10 transgene and containing both RNA transport elements exhibited almost no loss in titer in comparison to a corresponding vector encoding only GFP. The optimized Rev-independent gene delivery system was used for delivery of Rev M10 transgene into T-lymphocytes. Upon challenge in single round infection assays with HIV-1, the modified T-cells produced fewer virus particles than control cells expressing GFP. This Rev-free packaging system may prove useful for targeting the Rev-RRE-Crm1 nucleocytoplasmic RNA transport pathway for inhibiting HIV replication.

## Introduction

Gene delivery systems based on HIV-1 consist of packaging constructs (also referred to as helper constructs) and a gene transfer vector [1]. The packaging constructs encode virion structural proteins such as Gag/Gag-Pro-Pol or Env or regulatory proteins such as Rev and Tat. The gene-transfer vector provides the genomic RNA for encapsidation by the virus-like particle generated by the packaging constructs. The gene transfer vector lacks protein coding regions of the provirus but retains the necessary cis-sequences required for RNA transport, encapsidation, reverse-transcription, and integration. The gene-transfer vector also carries the transgene expression cassette.

The Gag and Gag-Pro-Pol proteins of HIV-1 are expressed from the full-length or unspliced mRNA. The mRNAs for the Env and accessory or regulatory proteins encoded by the virus are derived from the full-length mRNA by alternative splicing [2]. It follows that the *gag/pro-pol* region is defined as an intron for production of spliced mRNA for expression of the other HIV-1 proteins. Normally, in eukaryotic cells, intron-containing messages are retained in the nucleus and only completely spliced messages are allowed to exit into the cytoplasm. Thus, the *gag/pro-pol* coding region would be spliced out before transport of the mRNA from the nucleus to the cytoplasm thereby precluding the production of virus particles. The virus overcomes this checkpoint in cells through expression of the viral regulatory protein Rev. The Rev protein binds to a structured RNA element, the Rev-response element (RRE) present in the envelope coding region, and through its interaction with host proteins such as Crm1, engineers the exit of the *gag/pro-pol* containing message from the nucleus into the cytoplasm [3], [4]. Thus, HIV-1 based packaging constructs contain, in addition to the gag/pro-pol coding sequence, the HIV-1 RRE as well. Production of virus-like particles from such a construct requires coexpression of Rev [5]. The gene-transfer vector RNA, while lacking most of the gag/pro-pol coding region still retains 5′ and 3′ splice sites, and therefore requires the RRE in cis and expression of Rev in trans for production of high-titer vector stock [1], [5].

Simpler retroviruses, such as the Mason-Pfizer monkey virus (MPMV), do not code for regulatory proteins such as Rev. But they have to overcome the same checkpoint for expression of the viral Gag/Gag-Pro-Pol polyproteins. The MPMV contains a structured RNA element, the constitutive transport element (CTE). This element functions *in cis* and does not require coexpression of a viral Rev-like protein to effect transport and translation of intron-containing messages [6], [7], [8]. Instead, the CTE uses cellular proteins to perform a function analogous to Rev and RRE. The MPMV CTE can substitute for Rev and RRE in HIV-1 proviral clones as subgenomic constructs encoding Gag/Gag-Pro-Pol or Env [5], [6], [9], [10].

Intriguingly, HIV-1 and MPMV utilize distinct cellular RNA transport machinery for export of intron-containing messages. The RNA transport pathway mediated by Crm1 is used by Rev and RRE [11], [12], [13] while the MPMV CTE utilizes cellular mRNA pathway mediated by Nxf1(Tap)-p15(Nxt1). The host proteins involved in Rev-RRE RNA transport pathway include Crm1 and the RNA helicase DDX3 [14]. In contrast, the MPMV CTE has been shown to require host proteins, Tap (Nxf1), p15 (Nxt1), Sam68 [15], [16], [17] and the RNA helicase Dbp5 [14]. Sam68 may be involved in other aspects of HIV-1 gene expression including the Rev-RRE-Crm1 pathway [18], [19], [20], [21]. An HIV-1 packaging system based on MPMV CTE would not only allow one to target Rev function using dominant negative proteins or RNA based approaches, but also host proteins unique to the Rev-RRE-Crm1 RNA transport pathway [22].

To that end, we created HIV-1 packaging systems containing one or more copies of MPMV CTE in the packaging or gene transfer vector constructs. While the CTE-modified packaging systems were indeed Rev-independent, and consequently not susceptible to inhibition by a dominant negative Rev mutant, Rev M10, they provided less than optimal titers. We were able to rectify this defect by including the RRE of simian immunodeficiency virus (SIVmac239) in the CTE-containing gene transfer vectors. This novel vector system was used for delivery of Rev M10 into Jurkat T-cells to provide intracellular ‘immunization’ against HIV-1 replication.

## Results

### Increasing the number of CTE moieties in packaging constructs enhances particle production

**Figure 1:**
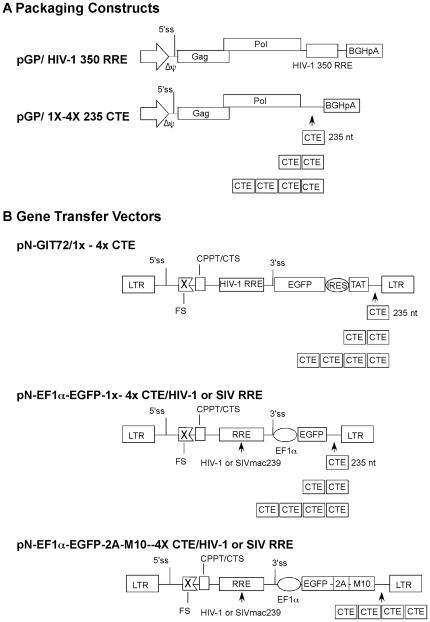
Schematic representation of packaging constructs (A) and gene transfer vectors (B). The packaging constructs (A) were created by inserting the *gag/pro-pol* coding region from pNL4-3 in pCDNA3 between the human cytomegalovirus immediate early promoter and the bovine growth hormone poly A signal (BGHpA). The plasmid pGP/HIV-1 350 RRE contains the HIV-1 RRE; while pGP/1-4xCTE contains either 1-, 2- or 4-copies of MPMV CTE, respectively. The RRE or CTE sequences were inserted between the *gag/pro-pol* coding sequence and the BGHpA signal. The gene transfer vectors (B) are derivatives of pN-GIT72 [27]or pN- EF1α-EGFP-WPRE[25]. They were modified to contain 1-, 2- or 4- copies of CTE. The HIV-1 RRE in pN- EF1α-EGFP-WPRE was replaced with the 1045 nt SIVmac239 RRE [24] to create pN- EF1α-EGFP-1x-4x CTE/SIV RRE. The vector pN- EF1α-EGFP-2A-M10- 4xCTE /SIV RRE is similar to pN- EF1α-EGFP-4xCTE/SIV RRE, but contains EGFP-2A-M10 fusion protein instead of only EGFP. FS: frame-shift mutation; 5′ss: 5′ splice site; 3′ss: 3′ splice site, ΔΨ : deletion in encapsidation signal; IRES: encephalomyocarditis virus internal ribosome entry site; 2A: foot and mouth disease virus 2A protease cleavage factor; CPPT/CTS: central polypurine tract/central termination sequence.

**Figure 2:**
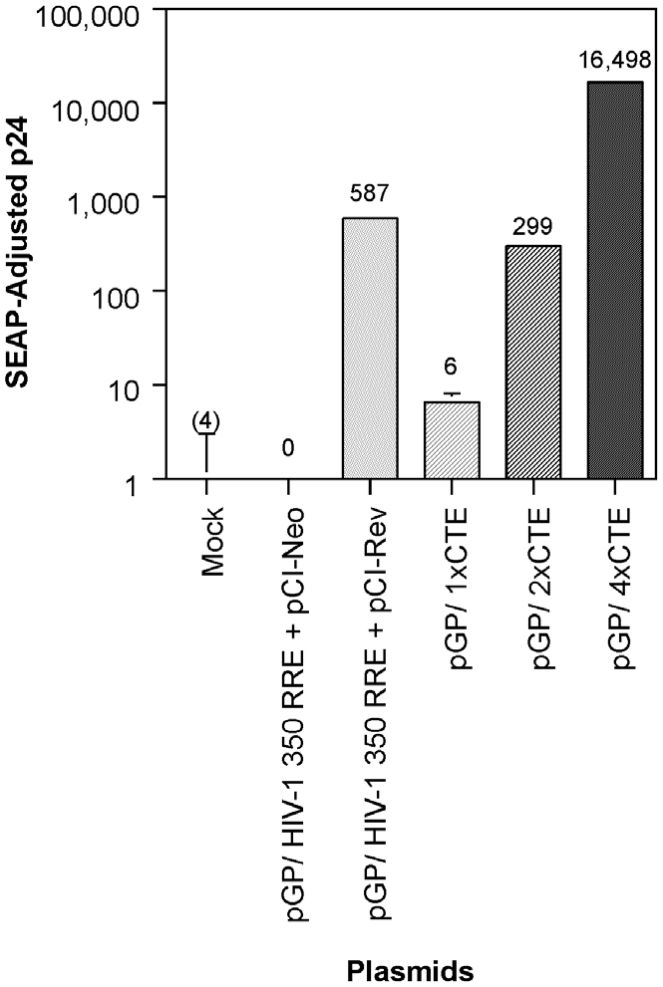
Particle production by packaging constructs with RRE or CTE HEK 293T were transfected with packaging plasmids pGP/HIV-1 350 RRE, pGP/1xCTE, pGP/2xCTE or pGP/4xCTE. The transfections with pGP/HIV-1 350 RRE, received either pCI-Neo or pCI-HIV Rev. The supernatants were harvested 72 hours post-transfection and assayed for HIV-1 capsid protein (p24) by ELISA and SEAP activity using a commercial kit. Mean SEAP-adjusted p24 levels are shown. Each experiment was carried out in duplicate. Error bar = 1 SD.

The HIV-1 Gag and Gag-Pro-Pol encoding packaging constructs, pGP/1xCTE, pGP/2xCTE and pGP/4xCTE, containing 1-, 2- and 4-copies of CTE (Figure 1A), respectively, were transfected individually into human embryonic kidney (HEK) 293T cells. For comparison, parallel transfections received pGP/HIV-1 350 RRE, a packaging construct containing RRE instead of CTE, together with pCI-HIV Rev, a Rev expression plasmid. Each transfection also received a secreted alkaline phosphate (SEAP) expression construct to normalize for transfection efficiency. The supernatants were harvested 72 h post-transfection and assayed for HIV-1 virion capsid protein (p24) by ELISA. The SEAP-adjusted p24 in the spent media of transfected cells is shown in Figure 2. As anticipated, significant amounts of p24 were detected in the media of cells transfected with pGP/ 350 HIV-1 RRE only in the presence of Rev. The CTE regulated Gag/Gag-Pro-Pol expression plasmids demonstrated different levels of p24 production depending on the number of CTE modules present in the construct. The p24 levels were approximately 50-fold higher for pGP-2xCTE than for pGP/1xCTE. Likewise pGP/4xCTE achieved 55-fold higher levels of p24 than pGP/2xCTE. The p24 levels observed for transfections with pGP/ HIV-1 350 RRE with pCI-HIV Rev were within 2-fold to that seen with pGP/2xCTE. Thus, pGP/4XCTE in the absence of pCI-HIV Rev achieved 28-fold higher levels of p24 than pGP/HIV-1 350 RRE with pCI-HIV Rev.

### Creation of a high-titer Rev-free packaging system requires modification of both packaging and gene transfer vector constructs with multiple copies of CTE

The encapsidation of genomic RNA from the gene transfer vector is likely to be determined by the efficiency of nucleocytoplasmic transport and by the localization of the vector or full-length RNA in the cytoplasmic compartment at the site of virion assembly [23]. We hypothesized that increasing number of CTE-modules in the gene transfer vector would result in enhanced cytoplasmic localization of the vector RNA in the absence of Rev. The enhanced encapsidation would manifest as improved vector titers. This premise was tested as described below.

The HEK 293T cells were transfected with the pN-GIT72 gene transfer vector containing 1-, 2- or 4-copies of CTE together with packaging constructs modified with 1-, 2- or 4-copies of CTE. The vector expressed the enhanced green fluorescent protein (EGFP) and a functional seventy-two amino acid Tat protein (Tat 72). One set of transfections received a plasmid, pCI-HIV Rev that encodes HIV-1 Rev, while the parallel set included the parent expression construct, pCI-Neo as negative control. All transfections also received a VSV-G expression plasmid (pMD.G) as well as a plasmid encoding SEAP. The resultant vector stocks were used for infection of naïve HEK 293T cells for determination of vector titer by flow cytometry [24].

**Figure 3:**
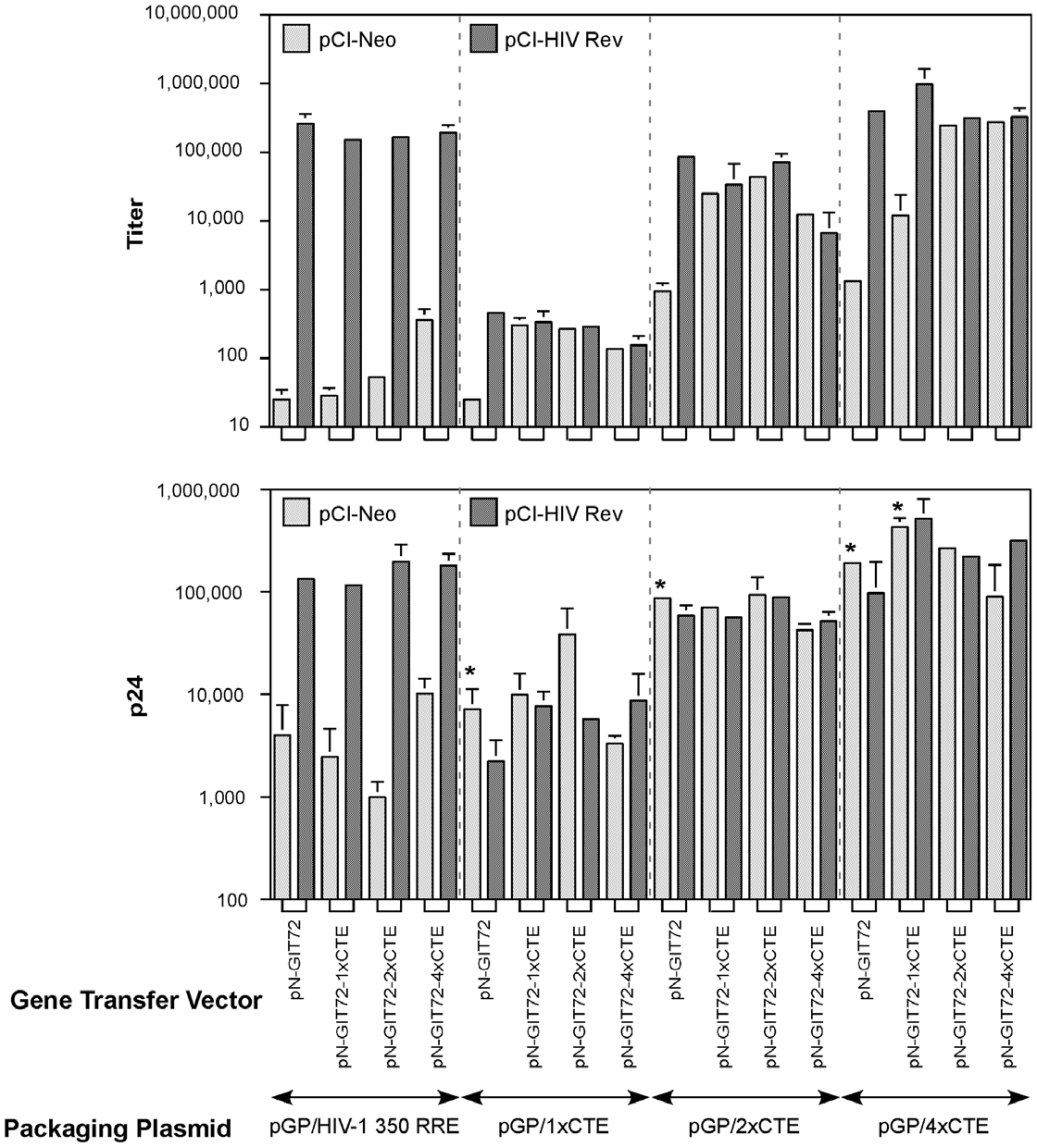
Virus stock production by various combinations of packaging and gene transfer vectors containing either HIV-1 RRE or one or more modules of MPMV CTE. HEK 293T cells were transfected with indicated packaging and gene transfer vectors. One set of transfections received pCI-Neo (speckled bars) and a parallel set of transfections received pCI-HIV Rev (cross-hatched bars). All transfections received VSV-G envelope expression construct and a SEAP expression construct. The supernatants were harvested 72 h post-transfection and used for infection of naïve HEK 293T cells. The virus titers (top panel) were determined by flow cytometry of infected cells as described in Materials and Methods and normalized for transfection efficiency against SEAP activity. The supernatants were also assayed for virus particle content by ELISA for HIV-1 capsid protein (p24). The SEAP-adjusted p24 values are shown in the bottom panel. Discordant results between the titers and the p24 levels are indicated by asterisks (*) above the corresponding bars. Each experiment was carried out in duplicate. Error bar = 1 SD.

The vector titers, normalized for transfection efficiency using SEAP levels, are shown in Figure 3A. Vector stocks generated using pGP/ HIV-1 350-RRE required coexpression of Rev even if the gene transfer vector contained one or more copies of CTE in addition to RRE. Thus, very low or background titers were observed in the absence of Rev but considerably higher levels were seen in its presence. When pGP/1xCTE, was used for packaging, all of the CTE-bearing vectors showed similar titers in the presence and absence of Rev. The only exception was the vector with only RRE and no CTE modules which showed diminished titers in the absence of Rev. As anticipated from the particle production experiments described in the previous experiment, the vector titers obtained with the packaging construct pGP/1xCTE were low in the presence and absence of a Rev. In contrast, packaging constructs pGP/ 2xCTE and pGP/4xCTE achieved titers that approached those of the control pGP/HIV-1 350 RRE even in the absence of Rev. This was particularly true of pGP/4xCTE, which generally provided higher titers than pGP/2xCTE. For the vector pN-GIT72 lacking CTE, Rev coexpression during virus stock production was still required with both pGP/2xCTE and pGP/4xCTE. These results demonstrated that for creating a Rev-independent packaging system both the packaging and gene-transfer vector constructs required modification with one or more copies of CTE.

To correlate vector titers with virus particles, the vector containing supernatants were tested for HIV –1 capsid (p24) antigen by ELISA. The p24 values are shown in Figure 3 B. For vector stocks produced with pGP/HIV-1 350 RRE, the titers were higher when transfections contained pCI-HIV Rev (Fig 3A). The titer differences between stocks produced in the presence or absence of pCI-HIV Rev could be explained by differences in the p24 levels. For the packaging plasmids containing one or more copies of CTE, presence or absence of Rev during virus production, did not affect p24 levels, but the titers were lower in the absence of Rev (indicated by ‘*’ in Figure 3 B). These results demonstrated that pCI-HIV Rev was necessary for packaging gene transfer vectors containing only RRE but no CTE modules.

### CTE-based packaging systems are resistant to Rev M10

**Figure 4:**
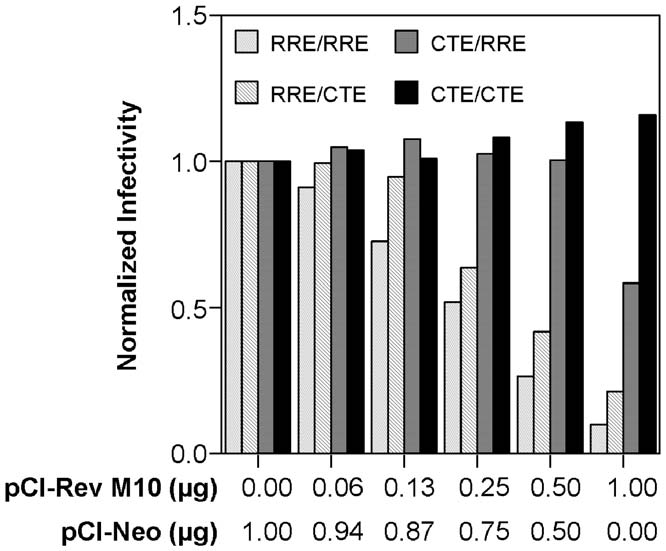
Effect of Rev M10 coexpression on vector production by packaging systems regulated by HIV-1 RRE, CTE or combinations thereof. Four different combinations of packaging and gene transfer vectors (RRE/RRE; RRE/CTE; CTE/RRE; CTE/CTE) were tested for production of vector stocks. The plasmid pCI-Rev M10 was used at the indicated amounts during vector stock production. The total amount of plasmid added was kept constant by using pCI-Neo as a filler plasmid. All transfections also received a VSV-G expression construct (pMD.G) and pCI-HIV Rev except for the packaging system (CTE/CTE) that used CTE in both packaging and gene transfer vector constructs. The vector titers were determined by infection of Jurkat T-cells. For each kind of packaging system, the titer obtained in the absence of pCI-Rev M10 was used for normalization. The different combinations were designated based on the transport element in the packaging construct and gene transfer vector. Each experiment was carried out in duplicate. Error bar = 1 SD. RRE/RRE: pGP/HIV 350 RRE and pN-GIT72; RRE/CTE: pGP/HIV 350 RRE and pN-GIT72-2xCTE; CTE/RRE: pGP/4xCTE an d pN-GIT72; CTE/CTE: pGP/4xCTE and pN-GIT72-2xCTE.

Having established a Rev-free packaging system, it was of interest to determine if this packaging system was susceptible to Rev M10, a dominant negative mutant of wild-type Rev. To this end, we produced vector stocks, using the CTE-based packaging system or the HIV-1 RRE/Rev based one, together with increasing amounts of pCI-Rev M10. The total amount of DNA added was kept constant by using pCI-neo, the parent plasmid for creating pCI-Rev M10, as a ‘filler’. We also tested the effect of pCI-Rev M10 on reciprocal or combination packaging systems in which either the packaging or the gene transfer vector contained CTE while the other construct was regulated by RRE and Rev. For the Rev-RRE based or the combination packaging systems, pCI-HIV Rev was also included during vector stock production. To allow comparison among the different packaging systems, the vector titers for a particular packaging system were normalized to the titer obtained in the absence of Rev M10 for that system. The results are shown in Figure 4. As anticipated, for the packaging system regulated by Rev and RRE, cotransfection with pCI-Rev M10 resulted in a dose-dependent decrease in vector titer. In contrast, the packaging system with CTE in both gene transfer vector and packaging construct was completely resistant to the inhibitory effects of Rev M10 at the dosages tested. The reciprocal or combination packaging systems exhibited intermediate phenotypes.

### Diminished titers of CTE-based packaging systems with gene transfer vectors containing internal promoters

We next wished to determine how CTE modules affected titers of vectors with internal promoters. For this purpose, we chose the previously described vector expressing EGFP under control of an internal EF1α promoter [25]. The gene transfer vector, pN-Ef1α-EGFP-WPRE, was modified by replacing the woodchuck post-transcriptional regulatory element downstream of the EGFP gene with 1-, 2- or 4-copies of CTE. The gene transfer vectors also contained the HIV-1 RRE (Figure 1 B).

We hypothesized, as with the pNGIT72 based vector constructs, that a) the packaging/encapsidation efficiency of genomic RNA from the gene transfer vector would be determined by the efficiency of nucleocytoplasmic transport and colocalization of the vector RNA in the appropriate cytoplasmic compartment at the site of virion assembly, and b) that increasing number of CTE modules would render the packaging system Rev independent. We tested this premise as follows.

The HEK 293T cells were transfected with pN-EF1α-EGFP-WPRE or vectors containing 1-, 2- or 4-copies of CTE together with pGP/4xCTE. One set of transfections received the plasmid pCI-HIV Rev that encodes HIV-1 Rev while a parallel set of transfections received the parent expression construct, pCI-Neo. All transfections also received a VSV-G expression plasmid (pMD.G), a Tat expression construct (pCMVtat) as well as a plasmid encoding secreted alkaline phosphatase (SEAP). The resultant vector stocks were used for infection of Jurkat-T cells for determination of vector titer.

**Figure 5:**
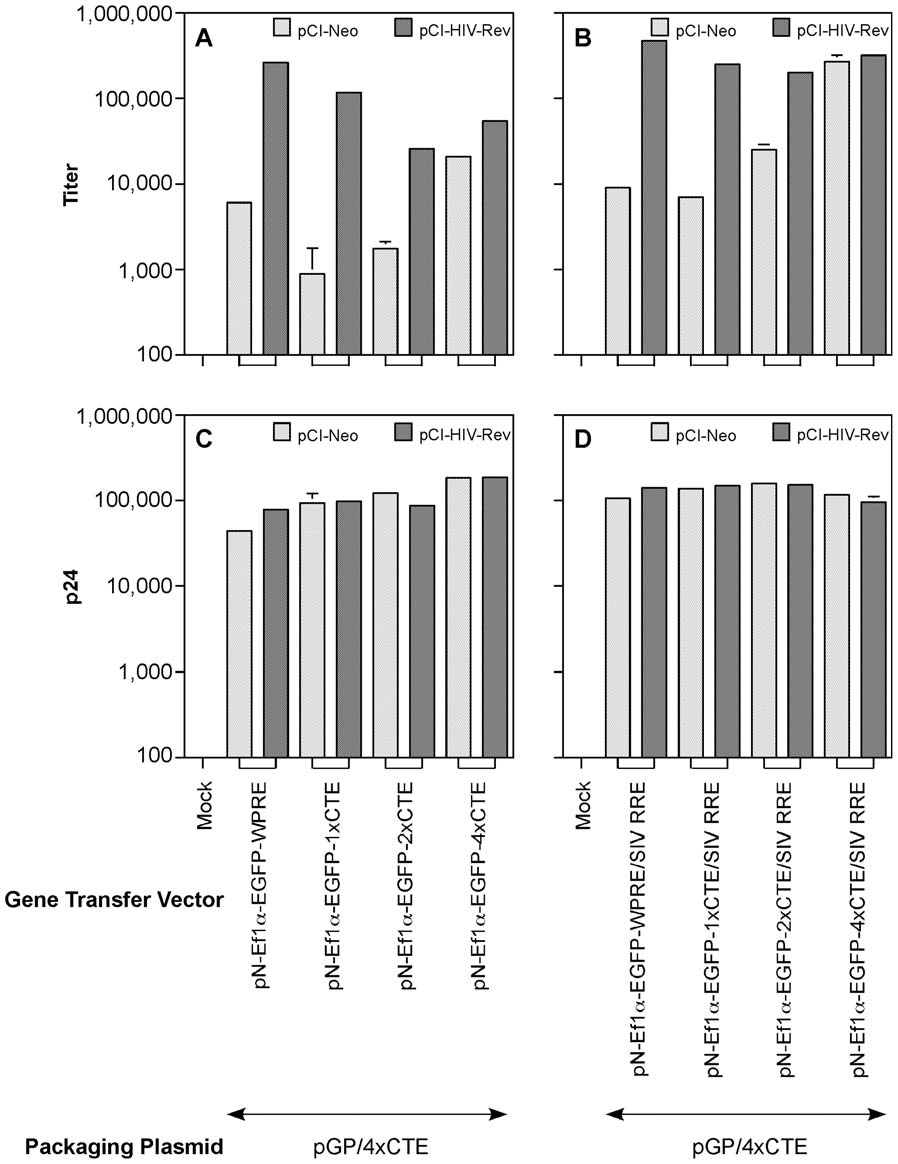
Packaging of gene transfer vectors containing CTE or both CTE and SIVmac239 RRE by pGP-4xCTE. The vectors pN- EF1α-EGFP-WPRE, pN- EF1α-EGFP-1xCTE, pN- EF1α-EGFP-2xCTE, and pN- EF1α-EGFP-4xCTE (A and C) and their corresponding SIV RRE containing vectors (B and D) were individually packaged using pGP/4xCTE in HEK 293T cells as described in Materials and Methods. One set of transfections received pCI-Neo (speckled bars) and a parallel set received pCI-HIV Rev (cross-hatched bars) The SEAP-adjusted titers are shown in panels A and B while the SEAP-adjusted p24 levels are shown in panels C and D. Each experiment was carried out in duplicate. Error bar = 1 SD.

The vector titers are shown in Figure 5 A. The results indicate that the titers were higher in the presence of Rev than in its absence for the pN-EF1α-EGFP-1xCTE and pN-EF1α-EGFP-2xCTE vectors. The difference was greater for pN-EF1α-EGFP-1xCTE than pN-EF1α-EGFP-2xCTE. Even with four copies of CTE, the vector pN-EF1α-EGFP-4xCTE exhibited a partial Rev-dependency. This difference was statistically significant (p<0.05; Table S1 ). Thus, while the results were similar to the pNGIT72 based vectors in exhibiting decreasing Rev-dependence with increasing number of CTE modules, complete Rev-independence was not seen even with four copies of CTE in the gene transfer vector. To determine if the difference between titers of vectors packaged in the presence or absence of Rev was due to differences in particle production the HIV-1 capsid protein content in the vector stocks was measured by p24 ELISA. The results (Figure 5 C) showed that all transfections achieved comparable amounts of p24 in the presence and absence of a Rev-expression construct. The difference in p24 levels were within 2.4 fold of the p24 value for the transfection with control vector containing only HIV-1 RRE while differences in the titer dwere much greater. These data suggested that the differences in titers could only be attributable to the Rev-independence of the gene transfer vectors.

Interestingly, the overall vector titers were reduced with increasing copies of CTE. Thus, as the number of CTE modules increased, the titer of the vector decreased even in the presence of Rev (Figure 5 A). The diminished titers were statistically significant (p value<0.05; Table S1).We addressed this observation by modifying the vector backbone as described below.

### Replacement of HIV-1 RRE with that of SIVmac239 restores titer of vectors containing multiple copies of CTE

We previously demonstrated that the SIV RRE could substitute efficiently for the HIV-1 RRE in both packaging and gene transfer vectors [24]. During the course of those studies we observed that SIV RRE containing vectors displayed slightly higher basal titers even in the absence of Rev. Moreover, they had the unexpected property of rendering the packaging system partially refractory to the inhibitory effects of Rev M10. We hypothesized that combining both the SIV RRE and MPMV CTE would improve vector titers without altering the phenotype of resistance to Rev M10. To this end, we created a series of HIV-1 vectors based on pN-EF1α-EGFP-WPRE containing the 1045 nt SIVmac239 RRE in place of the HIV-1 RRE, together with one or more copies of CTE in place of WPRE (see Figure 1 B). Each of the vectors was used for production of virus stocks with the packaging plasmid pGP/4xCTE in the presence and absence of pCI-HIV Rev. The resultant SEAP-adjusted titers are shown in Figure 5 B and indicate that replacement of HIV-1 RRE with that of SIVmac239 RRE in CTE containing vectors restored vector titers to near normal levels. The SIV RRE modified vectors with one or two modules of CTE were still responsive to Rev in that titers were higher in the presence of Rev than in its absence. However, the titers in the absence of Rev were much higher for the vectors with the SIV RRE than the corresponding vectors with HIV-1 RRE. Again, these differences could not be attributed to differences in particle production since results of p24 ELISA showed comparable levels of the virus capsid protein in the supernatants (Figure 5 D). The p24 levels were within 0.4 fold of the value of the transfection with the vector with SIVmac239 RRE and no CTE and could not be distinguished statistically (p>0.05). The SIV RRE modified vector with four copies of CTE, pN-EF1α-EGFP-4xCTE/SIV RRE, achieved similar titers both in the presence of Rev and in its absence. These titers were statistically indistinguishable (p value>0.05) Thus, replacing HIV-1 RRE with SIV RRE in CTE-containing gene transfer vectors significantly improved titers of CTE based packaging systems.

### Efficient delivery of Rev M10 into T-cells by gene-transfer vectors containing both CTE and SIVmac239 RRE

**Figure 6:**
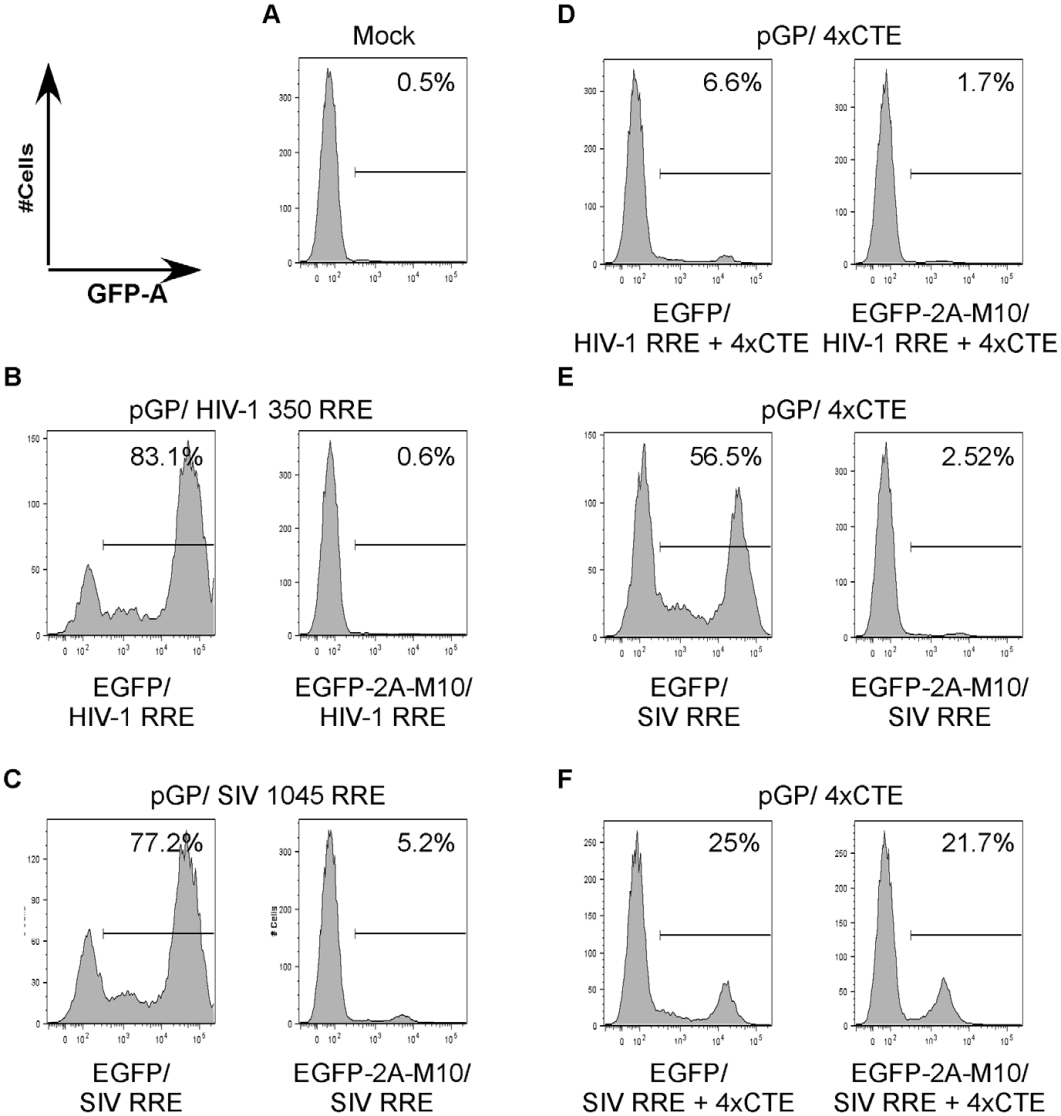
Flow cytometry analysis of Jurkat T-cells transduced by HIV-1 vectors encoding EGFP or EGFP-2A-Rev M10. The histograms show cell number along the Y-axis and EGFP expression along the X-axis. The packaging constructs used are indicated above the histograms, while the transgene and the transport elements in the gene transfer vectors are shown below. The same packaging construct was used for each pair of gene transfer vectors. The percentage EGFP positive population, as determined by marker/gate M1, is also shown. Representative data from an experiment carried out in duplicate.

**Figure 7:**
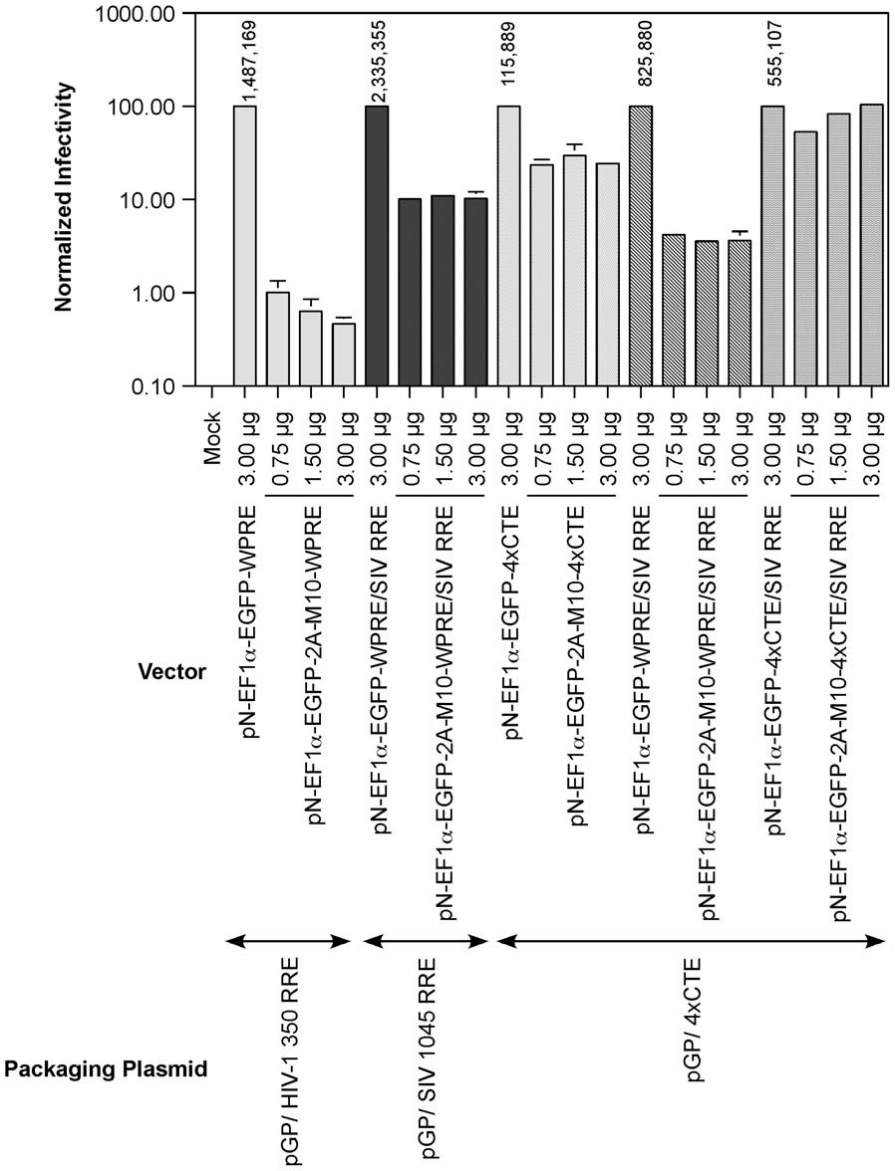
Comparison of packaging systems for delivery of Rev M10 into Jurkat T-cells. Packaging plasmids pGP/HIV-1 350 RRE, pGP/SIV 1045 RRE and pGP/4xCTE were used to produce virus stocks with indicated gene transfer vectors. The virus stocks were used for infection of Jurkat T-cells and vector titer was estimated. The titer of each Rev M10 encoding vector, was normalized to that obtained with the corresponding control vector expressing only EGFP. The SEAP-adjusted titers for the control vectors are indicated above the corresponding bars. The Rev M10 encoding vectors were tested at three different amounts as shown. Each experiment was carried out in duplicate. Error bar = 1 SD.

Encouraged by these results, we created gene transfer vectors containing four modules of CTE and encoded both EGFP and the transdominant Rev mutant , Rev M10. The two transgenes were expressed under control of the EF1α promoter enhancer elements as a single fusion protein, EGFP-2A-Rev M10, separated by the 2A protease cleavage factor derived from foot and mouth disease virus (FMDV). This configuration allows equimolar expression of both EGFP and Rev M10. The FMDV 2A cleavage factor ensures the release of the Rev M10 moiety by proteolytic cleavage subsequent to the synthesis of the fusion protein. Since SIV RRE containing vectors have also exhibited resistance to Rev M10, we created vectors that contained both SIVmac239 RRE as well as four copies of CTE. The control vectors lacked CTE but contained RRE from either HIV-1 or SIVmac239. The vectors pN-EF1α-EGFP-2A-M10-4xCTE, pN-EF1α-EGFP-2A-M10-WPRE/SIV RRE and pN-EF1α-EGFP-2A-M10-4xCTE/SIV RRE were individually packaged using pGP-4xCTE. Each of the above vectors was compared to control vectors encoding only EGFP for generation of vector stocks. Three different amounts of each vector were tested for generation of stocks to determine if this could influence final titers. We compared each of the above packaging combinations with the traditional packaging system based on HIV-1 RRE and Rev and also our recently described SIV RRE based packaging system[24]. The vector stocks were used for transduction of Jurkat T-cells to determine vector titers. A representative flow cytometry analysis is shown in Figure 6. The vector titers derived from flow cytometry are shown in Figure 7. To allow comparison between the different packaging systems, the titers were normalized to that of the control vector encoding EGFP alone for each packaging system.

Consistent with our earlier results [24], attempts at packaging a vector encoding EGFP-2A-M10, pN-Ef1α-EGFP-2A-M10-WPRE, using a HIV-1 Rev/RRE based packaging system, resulted in a dose-dependent decrease in vector titer between 97- to 215-fold in comparison to control vector, pN-Ef1α-EGFP-WPRE, that encoded only EGFP. In contrast, a pure SIV RRE/HIV-1 Rev based packaging system consisting of pGP-SIV 1045 RRE and pN-EF1α-EGFP-2A-M10-WPRE/SIV RRE demonstrated a titer decline of 9- to 10- fold with respect to pN-EF1α-EGFP -WPRE/SIV RRE. The CTE-based packaging system displayed even less sensitivity to encoded Rev M10. Thus the titers of the vector encoding Rev M10 was decreased by only 5- to 6-fold in comparison to the control vector encoding only EGFP. When we attempted to package an SIV RRE containing Rev M10 encoding vector with pGP/4xCTE, the titers were reduced 24- to 28-fold. In contrast, a vector containing both SIV RRE and CTE, pN-EF1α-EGFP-2A-M10-4xCTE/SIV RRE, when packaged with pGP/4xCTE displayed no diminution of titer from the control vector encoding EGFP alone, pN-EF1α-EGFP-4xCTE/SIV RRE. Thus, combining four copies of CTE with SIV RRE in the gene transfer vector encoding Rev M10 allowed production of stocks with very little reduction of titer (0.9 to 1.9-fold).

### Jurkat T-cells transduced with EGFP-2A-Rev M10 encoding vectors release fewer particles than cells transduced with control vector encoding only EGFP

**Figure 8:**
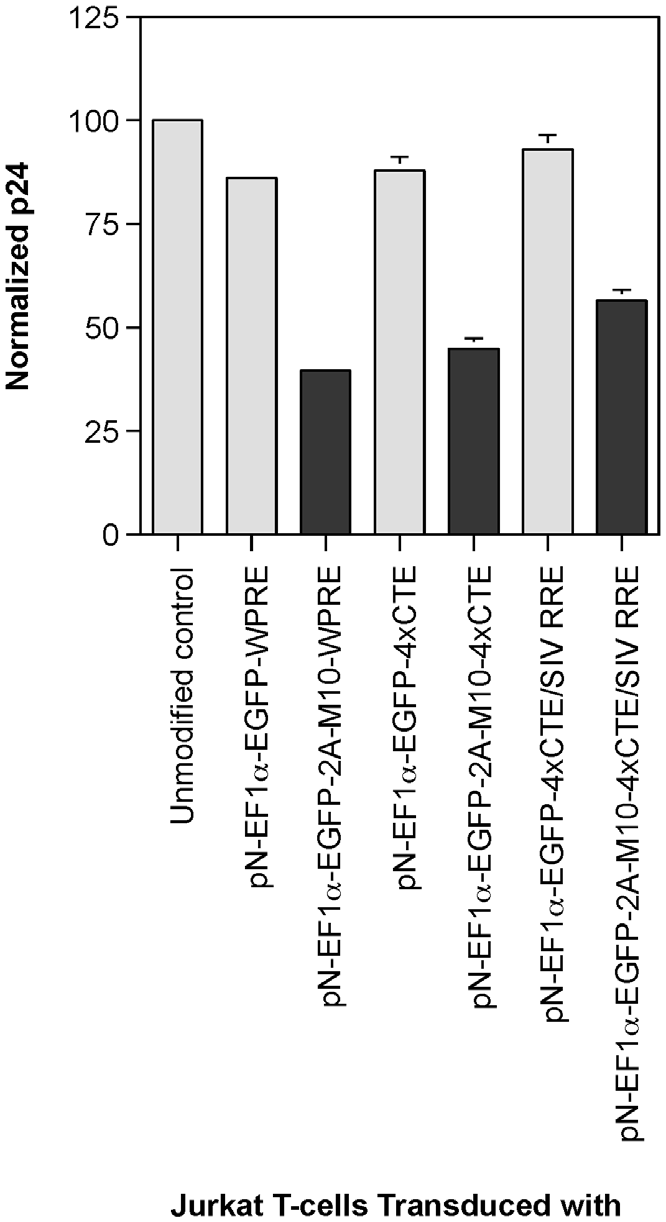
Virus particle production by gene modified Jurkat T-cells challenged with replication defective HIV-1. Jurkat T-cells, either unmodified or transduced with the indicated vectors encoding either EGFP (light grey) or EGFP-2A-M10 (dark grey), were challenged with replication defective HIV-1 pseudotyped with VSV.G as described in Materials and Methods. The HIV-1 capsid protein released into the supernatant of the infected cells was measured by p24 ELISA. The results were normalized to the p24 released from infected but unmodified control cells. Each experiment was carried out in duplicate. Error bar = 1 SD.

Jurkat T-cells transduced with each of the different vectors described in the previous experiment were sorted to greater than 90% purity. The EGFP expression levels were comparable between each of the sorted populations. Each pool of cells derived from the transduction of one vector, were infected with a replication-defective pNL4-3 encoding mouse heat stable antigen (CD24). Following infection, the cells were extensively washed and returned to the wells and placed in the incubator. The culture supernatants were harvested and assayed for HIV-1 capsid antigen by p24 ELISA. The results of p24 assay on day 8 are shown in Figure 8 and indicate that p24 levels, normalized to levels found in unmodified Jurkat T-cells, were decreased in each of the Rev M10 encoding cells in comparison to the control cells encoding only EGFP. Pair-wise comparison of the data showed that these differences were statistically significant (p value<0.05). Thus, modification of vectors with four copies of CTE or both SIV RRE and CTE did not adversely affect anti-HIV-1 activity of encoded Rev M10. The differences could not be attributed to differences in levels of infection since cell surface staining for HSA (CD24) encoded by the challenge virus revealed similar percentage of infected cells for M10 vector transduced cells and the corresponding control cells transduced with EGFP encoding vector counterpart (data not shown).

## Discussion

In this study, we sought to create a high-titer Rev-free HIV-1 packaging system based on the MPMV CTE. Such a packaging system should prove useful not only for targeting HIV-1 Rev, but also find use in targeting cellular cofactors unique to the Rev-RRE-Crm1 nucleocytoplasmic RNA transport pathway. Thus the CTE-based packaging system might be useful for targeting cellular cofactors such as the RNA helicases DDX3 and/or DDX1 purported to be unique to the Rev-RRE-Crm1 transport pathway[14], [26]. To that end, we created packaging systems containing one or more copies of MPMV CTE. We found that using a single copy of CTE in packaging and gene transfer vector resulted in a packaging system that was Rev-independent, but provided somewhat low titers attributable to lower levels of p24 produced by the packaging construct, pGP/1xCTE. Adding 2- or 4- copies of CTE in both the packaging and gene transfer vectors allowed the generation of stocks with much higher vector titers. In general, Rev-dependence decreased with increasing number of CTE modules in the vectors. The most likely explanation is that with insufficient numbers of CTE modules fewer of the full-length vector RNA were available in the cytoplasm for encapsidation into the particles. Addition of Rev with such vectors presumably improved nucleocytoplasmic transport of full-length genomic/vector RNA resulting in enhanced titers.

A surprising observation was that while increasing the number of CTE modules in the gene transfer vector, particularly those containing an internal promoter for directing expression of the transgene, rendered the packaging system Rev-independent, the overall titers tended to decrease (Figure 5). The increased particle production by packaging constructs regulated by multiple copies of CTE may be due to either a positive effect on transport, enhanced polysomal association or increased translation. In the case of the gene transfer vector which contained both HIV-1 RRE and CTE, addition of Rev lead to an increase in titer, but the titer did not approach that of the control vector containing only HIV-1 RRE. This increase in titer can be attributed to enhanced transport via the Rev-RRE-Crm1 transport pathway. Since the titer was not completely restored in comparison to the control vector with no CTE moieties but only HIV-1 RRE, the CTE appeared to be having a negative impact on the availability of the vector RNA for encapsidation. In the absence of Rev, the transport of RNA containing both RRE and CTE would be dependent on Tap-Nxf1 pathway. However, even for vectors with four copies of CTE, the titer did not approach that of the control vector regulated by Rev and RRE. One possible explanation for low titers with vectors containing multiple copies of CTE could be that the vector RNA was sequestered in the ‘wrong’ cytoplasmic compartment, such as polysomes, making the RNA less available for packaging by the assembling virion. Alternatively, the RNA may be less stable. We hope to address these possibilities in future studies that will address the stability, subcellular distribution and cytoplasmic fate of packaging and gene transfer vector RNAs in producer cells.

In our previous study, we noticed that HIV-1 RRE could be replaced with that of SIVmac239 RRE without a drop in titer [24]. Moreover, this system displayed considerable tolerance to the inhibitory effects of Rev M10. The SIV RRE based packaging system also exhibited slightly higher basal titers in the absence of Rev during virus stock production. We hypothesized that replacing the HIV-1 RRE with that of SIVmac239 RRE in CTE-containing gene transfer vectors might improve vector titers even in the absence of Rev during vector stock production. Consistent with this premise, the titers of vectors with four copies of CTE could be improved by replacing the HIV-1 RRE in the gene transfer vectors with that of SIVmac239 RRE (Figure 5). It is presently not clear how replacing HIV-1 RRE with that of SIVmac239 improved titers. The HIV-1 RRE sequence contains a 3′ splice acceptor site, as does the one from SIVmac239. It is not clear how critical this splice acceptor site is for the vector. While we have not created a CTE-containing vector lacking the RRE, we have tested a vector with multiple mutations in the RRE that renders it non-functional with Rev[10] with no positive impact on the titer. We are in the process of designing additional studies, as outlined above, to understand how SIV RRE improves titer of CTE containing vectors in a Rev-free packaging system.

To determine the suitability of the Rev-free packaging system for delivery of anti-Rev transgenes, we first tested the CTE-based packaging systems for their tolerance to the inhibitory effects of exogenously expressed Rev M10. As expected the CTE-based packaging system was indeed immune to Rev M10 expressed in trans during vector stock production. In contrast, the control packaging system containing the HIV-1 RRE and no CTE modules was exquisitely sensitive to inhibition by Rev M10 and exhibited a dose-dependent decrease in vector titer. Encouraged by these results, we created gene CTE containing transfer vectors encoding Rev M10 and the EGFP marker gene. For comparison we tested this vector system to our earlier packaging system containing SIVmac239 RRE. Both the CTE and the SIV RRE -based packaging system were found to be superior to the HIV RRE based one for production of Rev M10-encoding vector stocks. But the titers were much higher with the SIVmac239 RRE-based packaging system than the one based on CTE. By combining both CTE and SIV RRE in the same gene transfer vector, we were able to create a Rev-free packaging system that achieved high titers and proved useful for delivery of Rev M10 into T-lymphocytes.

There are several advantages to the packaging system described in this study compared to our previous one based on SIV RRE alone. The SIV RRE based packaging system requires Rev for production of vector stock thus signaling the utilization of the Crm1 RNA transport pathway. In contrast the CTE-based packaging system described in this study is Rev-independent and is expected to use the Nxf1/Tap-mediated RNA transport pathway. Although we, and others, have previously used the MPMV CTE in either packaging or gene transfer vectors, none of the packaging systems to our knowledge have achieved the efficiency of the present packaging system for production of high-titer vector stocks. This packaging system also exhibited remarkable resistance to the dominant negative mutant Rev M10 and consequently provided the highest titers of vector stocks encoding this transgene.

In a previous study we described Rev- free packaging cell lines based on MPMV-CTE and proposed that the CTE-based systems may be useful for delivery of the dominant negative Rev M10 [5]. Those packaging cell lines were restricted in their host-range due to the use of HIV-1 envelope for pseudotyping vector particles. Subsequently, we described packaging systems based on a transient transfection approach to produce vector stocks to improve the host range by use of amphotropic murine leukaemia virus envelope or VSV-G envelope glycoproteins [9], [27]. In those studies we suggested that the usage of dissimilar RNA transport elements could render the packaging systems safer. Since then, other groups have also used CTE in packaging systems with varying degrees of success. Mautino, et al, initially used CTE in gene transfer vectors, but the CTE in these vectors were positioned upstream of the transgene expression cassette [28], [29]. In subsequent studies, Mautino and coworkers used vectors with the CTE downstream of the transgene expression cassette[29], similar to what we used in our previous studies as well as in the present one. However, their group did not describe a completely Rev-free packaging system. The investigators also did not evaluate multiple CTE modules in the gene transfer vector or packaging constructs. More recently, Oh and coworkers described the creation of Rev-independent packaging systems based on multiple CTE modules [30]. These investigators also did not test their packaging system for delivery of anti-Rev transgenes. Our results are in general agreement with the observations of Oh and coworkers but also exhibit differences. Thus both studies show that increasing the number of copies of CTE in the packaging and gene transfer vectors can result in enhanced titers and also decrease Rev-dependency. We also noticed a decrease in overall titers with increasing CTE modules in our study that could be restored by using SIVmac239 RRE. The differences in the results of the two studies may reflect differences in vector configurations. Thus the vectors used in the two studies differed in the choice of internal promoters (EF1αpromoter in this study vs PGK in the study of Oh et al), the use of RRE from either HIV-1 or SIVmac239 in addition to the CTE modules in our study (no RRE in the CTE-containing vectors described by Oh et al.) and the location of the RRE with respect to the transgene expression cassette (upstream of transgene expression cassette in our study vs downstream in the study by Oh et al.). Furthermore, we demonstrated the utility of our Rev-free packaging system for delivery of any anti-Rev genes into T-lymphocyte cell lines. The present study therefore extends the studies of Oh and coworkers as well as our own earlier observations. Since, the HIV-1 RRE has been replaced with SIVmac239 RRE, our packaging system may prove useful for targeting other HIV-1 sequences such as the HIV-1 RRE, Rev[31] and/or Env using antisense [32], [33]or RNAi approaches[34]. The Rev-free packaging system could conceivably be used to target host factors that are unique to the Rev-RRE-Crm1 nucleocytoplasmic RNA transport pathway, such as RNA helicases, to thwart HIV-1 replication [35], [36].

## Materials and Methods

### Cell lines

Human embryonic kidney (HEK 293T) cell line was obtained from the American Type Culture Collection (Manassas, VA) (catalog number CRL-11268) and maintained in Dulbecco's modified Eagle's medium supplemented with 10% fetal bovine serum, 2 mM L-glutamine, penicillin (100 u/ml) and streptomycin (100 µg/ml).

Human T lymphocyte (Jurkat-T) cell line was also obtained from American Type Culture Collection (catalog number TIB-152) and maintained in RPMI1640 supplemented with 10% fetal bovine serum, 2 mM L-glutamine and 1.0 mM sodium pyruvate.

### Plasmids

#### Packaging constructs

pGP/HIV 350 RRE and pGP/SIV 1045 RRE have been described earlier [24]. Briefly, these constructs contain the *gag/pro-pol* coding region including the 5′ splice donor site of the molecular clone pNL4-3 positioned between the human cytomegalovirus immediate early promoter and the bovine growth hormone polyadenylylation site. The inserted sequence lacks the core packaging sequence present between the splice donor site and upstream of the *gag* AUG codon. The plasmid pGP/HIV 350 RRE contains the extended RRE of pNL4-3 [37] while pGP/SIV 1045 RRE contains a 1045 nt derived from SIVmac239. The packaging constructs pGP/1xCTE, pGP/2xCTE and pGP/4xCTE, contain one two or four copies of CTE derived from MPMV positioned downstream of the *gag/pro-pol* sequence. In contrast to our previously described CTE-containing packaging construct [9], the constructs in this study do not use the polyA signal in the CTE but that of bovine growth hormone (BGH). The plasmid pGP/1xCTE was constructed in several steps. First, the CTE from pN-FS-sCMVluc-CTE [9] was isolated using NotI and XhoI and ligated into the same sites of pCDNA3 to create pCDNA3-CTE. Next, the HIV-1 *gag/pro-pol* sequence was isolated from pgp [9] by first digesting with XbaI followed by repair using T4 polymerase and then digesting with NotI. The isolated fragment was inserted between EcoRV and NotI sites of pCDNA3-CTE to create pGP/1xCTE.

To create pGP/2XCTE, the MPMV CTE was isolated from pN-FS-sCMVluc-CTE using SalI and XhoI and inserted into the XhoI site of pGP/1xCTE to create pGP/2XCTE. To create pGP-4XCTE, first, pgp was cut with XbaI, repaired using T4 polymerase and then digested with NotI. The isolated sequence containing *gag/pro-pol* was inserted into pCDNA3 between EcoRV and NotI sites to create pCDNA3-GP. Next a fragment containing four copies of CTE was isolated from pN-GIT72-4xCTE (see below) using XhoI and SalI and inserted into the XhoI site of pCDNA3-gp to give pGP/4xCTE.

#### Gene-transfer vectors

To create pN-GIT72-1xCTE, the CPPT/CTS sequence from pN-GIT72/CPPT [38] was isolated using BssHII and BsaBI and used to replace the corresponding sequence in pN-GITC [27]. To create pN-GIT72-2xCTE, a NotI-XhoI fragment, containing EMCV-IRES and one copy of CTE, was isolated from pN-GIT72-1xCTE and ligated into pN-GIT72-1xCTE digested with NotI and SalI which cuts at the 5′ end of the CTE thus resulting in a vector containing two copies of CTE.

The vector pN-GIT72-4xCTE, was created using a similar cloning strategy using pN-GIT72-2xCTE. To create pN-EF1α-EGFP-1xCTE, the EGFP-IRES-Tat-WPRE cassette between BsabI and SalI sites in pNGI72-1xCTE was replaced with the BsabI and SalI fragment from pN-Ef1α-EGFP-WPRE 27. The vectors, pN-EF1α-EGFP-2xCTE and pN-EF1α-EGFP-4xCTE, were created using a similar cloning strategy using pN-GIT72-2xCTE and pN-GIT72-4xCTE. The vectors with both CTE and SIV RREs, pN-EF1α-EGFP-1xCTE/SIV RRE, pN-EF1α-EGFP-2xCTE/SIV RRE, pN-EF1α-EGFP-4xCTE/SIV RRE were created by replacing the BsabI and SalI fragment in pNGIT72-1xCTE, pNGIT72-2xCTE and pNGIT72-4xCTE with the ClaI and SalI fragment containing SIVmac239 RRE from pN-EF1α-EGFP-WPRE/SIV RRE.

The vectors encoding EGFP-2A-Rev M10 transgene, (pN- EF1α-EGFP-2A-M10-1xCTE, pN- EF1α-EGFP-2A-M10-2xCTE, pN- EF1α-EGFP-2A-M10-4xCTE) were created by replacing the BsabI and SalI fragment in pNGIT72-1xCTE, pNGIT72-2xCTE and pNGIT72-4xCTE with the BsabI and SalI fragment from pN- EF1α-EGFP-2A-M10-WPRE. To create vectors encoding EGFP-2A-Rev M10 and containing both CTE and SIV RRE (pN- EF1α-EGFP-2A-M10-1xCTE/SIV RRE, pN- EF1α-EGFP-2A-M10-2xCTE/SIV RRE, pN- EF1α-EGFP-2A-M10-4xCTE/SIV RRE), the BsaBI and SalI fragment in pNGIT72-1xCTE, pNGIT72-2xCTE and pNGIT72-4xCTE were replaced with the ClaI and SalI fragment containing SIVmac239 RRE from pN- EF1α-EGFP-2A-M10-WPRE/SIV RRE. Other constructs, pN-E EF1α-EGFP-WPRE, pN- EF1α-EGFP-WPRE/SIV RRE, pN- EF1α-EGFP-2A-M10-WPRE and pN- EF1α-EGFP-2A-M10-WPRE /SIV RRE, have been described previously [24].

### Vector stock production

Vector stocks were prepared by transient transfection of 293T cells by the CaPO_4_-method as previously described [39]. Briefly, 1×10^6^ cells were seeded into 6-well tissue culture plates one day prior to the transfection with the following plasmid cocktail: a packaging constructs (1.5 µg), a gene-transfer vectors (usually 3.0 µg), and a VSV-G envelope expression construct (pMD.G, 0.2 µg). A tat expression construct, pCMVtat [5] was used for those vectors that did not encode Tat. Other constructs such as pCI-Neo, pCI-HIV Rev were used as indicated. Most transfections also included a SEAP expression construct to normalize for transfection efficiency. The cell culture medium was replaced with fresh medium the day following the transfection. The virus –containing medium was harvested 48 to 72-hours later, clarified by low-speed centrifugation and either used immediately for infection or saved frozen at –80°C.

### Titration of vector stocks

This was done as previously described using either naïve 293T cells or Jurkat-T cells as targets. Briefly, 250,000 Jurkat T-cells were plated in 24-well plates on the day of the infection in 0.5 ml of medium containing 10 µg of polybrene/ml. An aliquot of vector stock was added to the medium. The following day, an additional one ml of fresh medium was added. The cells were harvested 48- to 72-hours later, washed and fixed overnight with 4% paraformaldehyde, pH 7.4. The cells were resuspended in PBS and the percentage of infected cells were determined by flow cytometry. The infectious or transducing units were calculated from the percentage of infected cells and the volume used for infection. For titer determination on 293T cells, the cells were seeded the previous day in 6-well plates. On the day of infection, the medium was replaced with fresh medium containing 10 µg/ml of polybrene to which an aliquot of virus stock was added. 48-72 hours later, the cells were harvested by trypsinization, fixed with formalin and the percentage of infected cells determined by flow cytometry as described above for Jurkat T-cells.

### Challenge experiments

Jurkat T-cells transduced with the indicated vectors were sorted to greater than 90% purity by using a cell sorter. The cells were infected with VSV-G pseudotyped pNL4-3-Vpr-Nef-HSA+ virus. The challenge virus was prepared by transient transfection of 293T cells as previously described [24]using pMD.G and pNL4-3.HSA.R-E- that encodes for pNL4-3-Vpr-Nef-HSA+ virus[40]. The following day, the cells were washed six times with fresh medium and resuspended in fresh medium. An aliquot of the medium was saved for p24 analysis. The spent medium was harvested every three days at which time the cells were also split at a ratio of 1∶5 or 1∶10. The harvested and cleared spent medium was assayed using p24-ELISA.

### Assays

HIV-1 p24 ELISA was done using a commercial kit obtained from PerkinElmer, (Massachusetts, USA). SEAP activity was measured by a chemiluminescent method with a commercial kit (Phopha-Light System) obtained from Applied Biosystems, (Massachusetts, USA).

## Supporting Information

Table S1 **Statistical analysis of titer differences between different vectors shown in** **Figure 5A and 5B** **using Student's t-test (two-tail).**
(DOCX)
