# Supplementary material for: Rev-Free HIV-1 Gene Delivery System for Targeting Rev-RRE-Crm1 Nucleocytoplasmic RNA Transport Pathway
Source: PLoS One. 2011 Dec 2;6(12):e28462. doi: 10.1371/journal.pone.0028462 (PMC3229575; doi:10.1371/journal.pone.0028462)
Supplement: Table S1 — Statistical analysis of titer differences between different vectors shown in Figure 5A and 5B using Student's t-test (two-tail). (DOCX) [file pone.0028462.s001.docx]

**Table S1.** Statistical analysis of titer differences between different vectors shown in Fig 5A and 5B using Student’s t-test (two-tail)

|  |  | **Packaging construct** | **RNA element in Gene transfer vector** | **Rev** | **Titer (IU/ml)** | **p values*** | | |
| --- | --- | --- | --- | --- | --- | --- | --- | --- |
| **Fig 5A** | **a** | pGP/4-xCTE | WPRE/HIV-1 RRE | - | 6,055 ± 434 | (a vs c) 0.04 | (a vs e) 0.02 | (a vs g) 0.01 |
|  | **b** | pGP/4-xCTE | WPRE/HIV-1 RRE | + | 262,346 ± 6,555 | (b vs d) 0.002 | (b vs f) 0.001 | (b vs h) 0.001 |
|  | **c** | pGP/4-xCTE | 1xCTE/HIV-1 RRE | - | 885 ± 885 | (c vs e) **0.45** | (c vs g) 0.01 |  |
|  | **d** | pGP/4-xCTE | 1xCTE/HIV-1 RRE | + | 117,351 ± 1,713 | (d vs f) 0.001 | (d vs h) 0.002 |  |
|  | **e** | pGP/4-xCTE | 2xCTE/HIV-1 RRE | - | 1,761 ± 343 | (e vs g) 0.01 |  |  |
|  | **f** | pGP/4-xCTE | 2xCTE/HIV-1 RRE | + | 25,779 ± 1,154 |  |  |  |
|  | **g** | pGP/4-xCTE | 4xCTE/HIV-1 RRE | - | 20,867 ± 1,698 | (g vs h) 0.01 |  |  |
|  | **h** | pGP/4-xCTE | 4xCTE/HIV-1 RRE | + | 54,689 ± 2,304 |  |  |  |
| **Fig 5B** | **i** | pGP/4-xCTE | WPRE/SIV RRE | - | 9,015 ± 1,119 |  |  |  |
|  | **j** | pGP/4-xCTE | WPRE/SIV RRE | + | 473,096 ± 18,276 | (j vs o) **0.07** | (j vs p) **0.06** |  |
|  | **k** | pGP/4-xCTE | 1xCTE/SIV RRE | - | 7,004 ± 274 |  |  |  |
|  | **l** | pGP/4-xCTE | 1xCTE/SIV RRE | + | 250,879 ± 11,232 |  |  |  |
|  | **m** | pGP/4-xCTE | 2xCTE/SIV RRE | - | 25,059 ± 3,819 |  |  |  |
|  | **n** | pGP/4-xCTE | 2xCTE/SIV RRE | + | 201,024 ± 7,358 | (n vs p) **0.07** |  |  |
|  | **o** | pGP/4-xCTE | 4xCTE/SIV RRE | - | 268,571 ± 52,659 | (o vs p) **0.50** |  |  |
|  | **p** | pGP/4-xCTE | 4xCTE/SIV RRE | + | 319,410 ± 32,880 |  |  |  |

* p values ≥0.05 are in **bold**.
